# Heterochromatin Dynamics

**DOI:** 10.1371/journal.pbio.0000014

**Published:** 2003-10-13

**Authors:** Tobias Straub

## Abstract

Heterochromatin is usually thought of as a stable and inactive region of the genome. Not so, according to a study published earlier this year

In 1928 the German botanist Emil Heitz visualised in moss nuclei chromosomal regions that do not undergo postmitotic decondensation (Heitz 1928). He termed these parts of the chromosomes heterochromatin, whereas fractions of the chromosome that decondense and spread out diffusely in the interphase nucleus are referred to as euchromatin. Further studies revealed that heterochromatin can be found in all higher eukaryotes, mainly covering regions with a low frequency of genes, such as pericentromeric regions and telomeres. Heitz proposed that heterochromatin reflects a functionally inactive state of the genome, and we now know that DNA in heterochromatic regions is less accessible to nucleases and less susceptible to recombination events. All these findings contributed to the current view that heterochromatin is a rigid nuclear compartment in which transcriptionally inactive regions of chromatin are densely packed and inaccessible to the transcription machinery (Grewal and Elgin 2002). This view was challenged earlier this year in two papers published back-to-back in *Science* (Cheutin et al. 2003; Festenstein et al. 2003).

Certain proteins are specifically associated with heterochromatin— notably, the family of heterochromatin protein 1 (HP1) (Eissenberg and Elgin 2000; Singh and Georgatos 2002). HP1 is thought to play a central role in creating a stable and inaccessible heterochromatic network by interacting with several other proteins, including histones, the major protein constituent of all chromatin. In particular, HP1 binds to the tail of the histone H3 when it has been modified by methylation of lysine 9. This histone modification is an important landmark of inactive chromatin regions.

In the two articles in *Science*, both groups generated cell lines stably expressing HP1 fused to green fluorescent protein (GFP) so that they could watch the behaviour of HP1 in living cells. Specifically, they used photobleaching techniques to study the in vivo mobility of HP1. In a defined region of a cell, fluorescently tagged proteins are bleached by a laser pulse. Recovery of fluorescence in the bleached area can then only occur if bleached molecules are replaced with unbleached molecules from regions outside the bleached area. The technique is called fluorescence recovery after photobleaching (FRAP) and provides information about the mobility and stability of the cellular structures and proteins. For HP1–GFP, the speed at which fluorescence recovers depends on how tightly it is bound within heterochromatic regions. Heitz (and many others) might have expected that heterochromatin-bound HP1 shows little turnover and therefore recovery should take place very slowly.

Cheutin et al. (2003) first demonstrated that the heterochromatic regions visualised with HP1–GFP are stable in shape for at least 2 h. By contrast, subsequent FRAP experiments revealed that HP1 proteins have a surprisingly high turnover rate in heterochromatic clusters as well as in regions the authors define as euchromatic (Figure 1). Recovery of 50% was reached after 2.5 s in heterochromatin and after 0.6 s in euchromatin. In contrast, for histone proteins, the structural protein components of chromatin, 50% recovery took more than 2 h (Kimura and Cook 2001). Cheutin et al. found that complete recovery of bleached HP1 took 5 s in euchromatin and 60 s in heterochromatin. Festenstein et al. (2003) report, however, that recovery only reaches 90% in euchromatin and 70% in heterochromatin. Incomplete recovery would point to an immobile population of HP1 that does not exchange rapidly. In fact, such a stable fraction could be indicative of a stable structural network made of a minor fraction of HP1 that could serve as a nucleation site for a more mobile fraction of HP1. In my opinion, this should be kept in mind, even if 100% recovery is observed. It might well be that a few stably associated HP1 molecules that remain undetected in FRAP studies exert an important structural function in heterochromatin formation. Consequently, this can be regarded as an important discrepancy between the two studies. Both studies also reported a number of other experiments in which the condensation state of chromatin was modified and was found to alter the mobility of HP1, such that relaxed condensation was associated with increased HP1 mobility.

**Figure 1:**
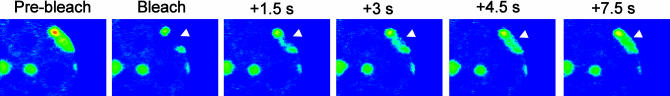
FRAP of HP1–GFP Reveals a Dynamic Association with Heterochromatin A fraction of a heterochromatic cluster (arrowhead) was bleached by a laser pulse, and recovery of fluorescence was monitored by time-lapse imaging. Images were kindly provided by Thierry Cheutin and Tom Misteli.

As discussed by the authors of both studies, several important conclusions can be drawn. In striking contrast to previous models, HP1 appears to be a very mobile molecule. The formation of heterochromatin appears not to be based on a stable oligomeric network of HP1 molecules. Furthermore, heterochromatin is accessible. There is no obvious constraint shielding these transcriptionally inactive compartments from factors residing outside. Given the rapid exchange of HP1 in heterochromatic clusters, any other soluble nuclear protein, such as a transcription factor, should be able to gain access, compete with silencing factors, and potentially activate genes located within heterochromatin. Taken together, heterochromatin appears to be a surprisingly dynamic compartment even though it forms morphologically stable entities. This dynamic situation could imply that heterochromatic silencing is not just a switch, but rather a continuous and active process.

Although the new work suggests that heterochromatin is more dynamic than was thought, some caveats remain. It is still possible that a stable “mark” of heterochromatin does exist. As I discussed above, this mark might be an undetected fraction of immobile HP1 molecules. In addition, one cannot exclude that HP1 is a downstream factor that is dynamically tethered to a stable binding site, the most likely candidate being the methylated histone tail. Perhaps the role of HP1 in heterochromatic silencing has simply been overinterpreted. The formation of facultative heterochromatin by X inactivation in mammals, for example, does not involve HP1 even though appropriate histone methylation marks are set. This indicates that heterochromatin formation does not always follow the same rules and suggests that our definitions of heterochromatin must be refined. In any case, it could well be that a silent state is marked by signals that have a slow turnover. In fact, histone methylation is believed to generate a quite stable “code” (Jenuwein and Allis 2001). Until this hypothesis has been tested in vivo, we should keep an open mind about the stability and dynamics of nuclear structure.

Several other nuclear compartments (spliceosomes, nucleoli) have also been proposed to consist of dynamic collections of components (Misteli 2001). Personally, I am intrigued by the fact that heterochromatin also might function as a steady-state association of molecules. Is the entire nucleus, the genome organization—irrespective of its functional state—in constant flux? Of course, such a situation would provide an appealing explanation for the plasticity of gene expression. On the other hand, dynamic control of gene expression complicates explanations of how established expression patterns are stably inherited. So far, genetic knockout experiments have been the most powerful tools to unravel the mechanisms of epigenetic regulation. Unfortunately, many of those investigations can only provide insight into the establishment of expression profiles. What happens if regulatory factors are knocked down after expression patterns are set up? Which signals will be erased and which ones will persist? I am working on the mechanism of dosage compensation in *Drosophila* (Lucchesi 1998). This process involves stable changes of chromatin structure, which leads to lasting effects on X-chromosomal gene expression. To examine the generality of the new results on heterochromatin, it will be important to find out whether the proteins involved in defining the X chromosome as an epigenetic compartment have the same dynamic behaviour as HP1. In addition, systematic knockdown of these factors after establishment of dosage compensation might disclose a hierarchy in epigenetic maintenance that is different from the one affecting establishment. 

## Abbreviations

FRAP: fluorescence recovery after photobleaching
GFP: green fluorescent protein
HP1: heterochromatin protein 1.
